# Molecular Action of Tamoxifen in the Ovaries of Rats with Mammary Neoplasia

**DOI:** 10.3390/ijms242115767

**Published:** 2023-10-30

**Authors:** Anna Nynca, Sylwia Swigonska, Tomasz Molcan, Brian K. Petroff, Renata E. Ciereszko

**Affiliations:** 1 Department of Animal Anatomy and Physiology, University of Warmia and Mazury in Olsztyn, Oczapowskiego 1A, 10-719 Olsztyn, Poland; reniac@uwm.edu.pl; 2 Laboratory of Molecular Diagnostics, University of Warmia and Mazury in Olsztyn, 10-720 Olsztyn, Poland; sylwia.swigonska@uwm.edu.pl; 3 Molecular Biology Laboratory, Institute of Animal Reproduction and Food Research, Polish Academy of Sciences, 10-243 Olsztyn, Poland; t.molcan@pan.olsztyn.pl; 4 Department of Pathobiology and Diagnostic Investigation, Michigan State University, East Lansing, MI 48910, USA; bpetroff@msu.edu

**Keywords:** tamoxifen, breast cancer, ovary, tumor-bearing rats, transcriptome, proteome

## Abstract

Tamoxifen (TAM) is a drug commonly used in patients with breast cancer. The anticancer effect of TAM occurs via its ability to antagonize estrogen-dependent growth of mammary epithelial cells. Previously, we demonstrated that TAM prevented the chemotherapy-induced loss of ovarian follicular reserves in both cancer-free rats and rats with cancer. Such follicular loss is a main cause of infertility in young women treated for cancer. The current study was undertaken to discover the molecules and intracellular pathways involved in the action of TAM in the ovaries of rats with mammary tumors. To meet this goal we used transcriptomic (RNA-Seq) and proteomic (2D-DIGE/MS) approaches. TAM inhibited the expression of genes and lncRNAs involved in ovarian steroidogenesis. Moreover, TAM altered the expression of genes related to primordial follicle activation or arrest. In addition, proteomic screening indicated the importance of basic metabolic processes in the ovarian actions of TAM. Although simple extrapolation of these data to humans is not possible, the results of this study emphasize the need to explore the ability of TAM to affect ovarian function in women undergoing cancer treatment.

## 1. Introduction

Tamoxifen (TAM) is a selective estrogen receptor modulator (SERM) commonly used as a drug in patients with hormone-dependent breast cancers [1], known to significantly improve their overall survival rate [2]. The therapeutic effects of TAM are due to the inhibition of estrogen-regulated pathways resulting from its ability to compete with estradiol (E2) for the estrogen receptor (ER) ligand-binding domain [3]. Following binding, TAM induces conformational changes in ER, blocking interaction of ER with coactivators [4] and thereby inhibiting ER-dependent proliferation of cancer cells. Tamoxifen can also affect breast cancer cells indirectly by modulating the concentration of cytokines and growth factors with anticancer properties [5]. The lipophilicity of TAM and its extensive binding to serum proteins prolong TAM action in target tissues [6].

Despite its antiestrogenic activity in breast cancer, TAM may exert estrogenic effects in other tissues (e.g., bones, cardiovascular system, reproductive tract) via the activation of ER. In the uterus, TAM has rare adverse effects such as endometrial carcinoma and uterine sarcoma related to estrogenic properties [7]. In addition, an increased risk of ovarian hyperstimulation, the development of ovarian cysts and supraphysiological levels of serum E2 were reported in TAM-treated women with breast cancer [8,9,10]. During the follicular phase, TAM was suggested to inhibit E2 negative feedback on the hypothalamic–pituitary axis, leading to increased pituitary secretion of FSH and LH and the development of multiple follicles. In other phases of the reproductive cycle, TAM may suppress the preovulatory LH surge leading to the formation of large follicular cysts [11]. Direct effects of TAM on granulosa cells followed by the enhanced serum E2 level were also suggested [12].

Tamoxifen also affects ovarian follicles in rodents with effects that depend on the stage of follicular development and experimental design. Tamoxifen was shown to promote primordial follicle activation [13] and increase the number of primordial follicles [14] as well as the number of atretic and cystic follicles [15,16]. On the other hand, TAM decreased the number of healthy secondary and antral follicles and corpora lutea [14,15]. Previously, we demonstrated that TAM did not affect the number of primordial and primary follicles in ovaries of mammary-tumor-bearing rats [17]. These data were consistent with antioxidant and apoptosis-related effects of TAM in the ovary [18,19]. There is increasing clinical interest in the effects and side effects of TAM in premenopausal women; it is therefore imperative to understand the molecular mechanism of TAM action in the ovary.

Our group [17,20] and other researchers [21] have shown that TAM has a protective effect on the ovaries of animals undergoing chemo- or radiotherapy. The current study, performed on TAM-treated rats with mammary tumors, was undertaken to recognize which molecules and which intracellular pathways may be involved in the action of TAM in the ovary. To meet this goal we used both transcriptomic (RNA-Seq) and proteomic (2D-DIGE/MS) approaches.

## 2. Results

### 2.1. Effects of TAM on the mRNA Expression Profile of Rat Ovaries

**Figure 1:**
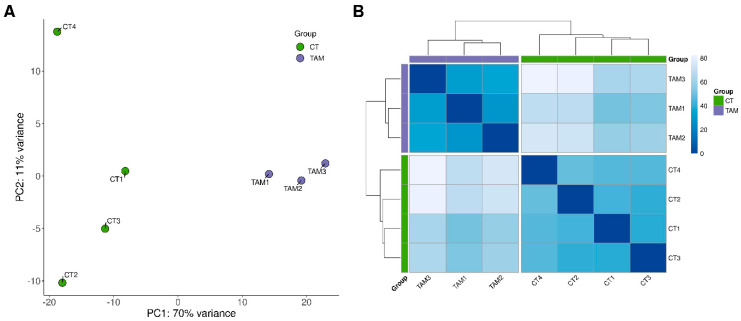
Graphical representation of (**A**) the first (PC1) and second (PC2) principal components (PC) (**B**) and samples’ distance matrix affecting lncRNAs expression pattern of rat ovaries.

The sequencing data from the current study were submitted to the BioProject database under accession number PRJNA640997. After removing reads containing adapters and low-quality reads, the remaining high-quality reads were mapped to the Ensembl rat genome (mRatBN7.2; Ensembl release 107). The number of reads aligned to the reference genome ranged from 34.8 to 38.1 million per sample, and an average of 95.4% of the reads were mapped to unique locations. The total number of genes expressed in the ovaries of tumor-bearing rats of all examined samples ranged from 19,805 to 20,231 (Table S2). The results of both the principal component analysis (PCA) and the analysis of distance matrices revealed a high level of similarity between the biological replicates within the control and TAM-treated rat groups (Figure 1).

**Figure 2:**
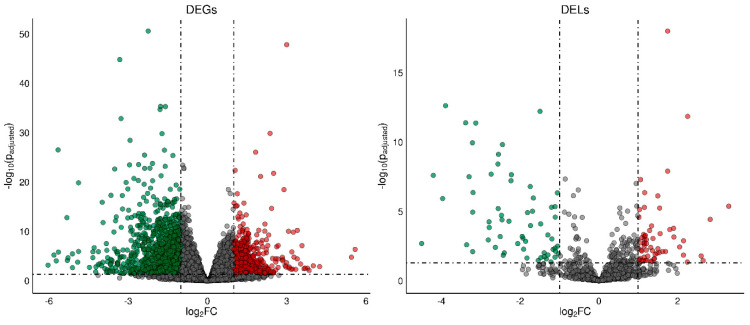
The volcano plot presenting differentially expressed genes (DEGs; *p*−adjusted ≤ 0.05 and log2FC ≥ 1.0 or log2FC ≤ −1.0) and lncRNAs (DELs; *p*−adjusted ≤ 0.05 and log2FC ≥ 1.0 or log2FC ≤ −1.0) in the ovaries of TAM−treated rats vs. untreated rats. DELs and DEGs are represented by multicolored circles, where red color is up−regulated DELs/DEGs and green down−regulated DELs/DEGs. Gray circles represent all lncRNAs or genes identified in the ovaries with no significant changes.

**Figure 3:**
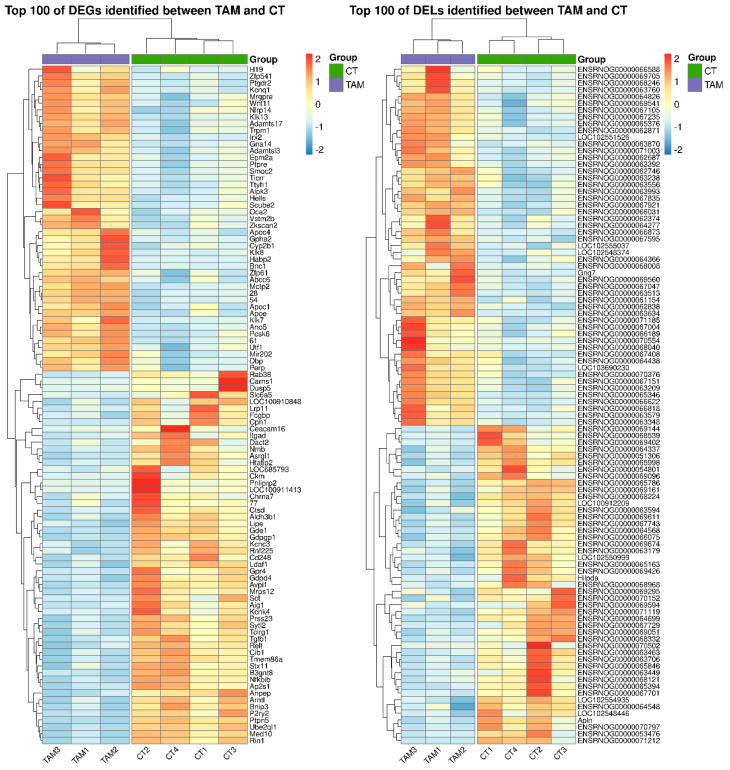
Heatmap of the top 100 differentially expressed genes (DEGs; *p*−adjusted ≤ 0.05 and log2FC ≥ 1.0 or log2FC ≤ −1.0) and lncRNAs (DELs; *p*−adjusted ≤ 0.05 and log2FC ≥ 1.0 or log2FC ≤ −1.0) demonstrated in the ovaries of rats treated with TAM. The expression values are presented as Z−score calculated from raw counts for each sample. The color scale of the heatmap shows the expression level where red blocks represent up− and pale blue blocks down−regulated genes or lncRNAs.

A total of 1788 differentially expressed genes (DEGs) were identified in the current study (Table S3). A total of 1084 of DEGs were down-regulated and 704 were up-regulated in rat ovaries of the TAM group compared to those of the control group. The distribution of DEGs (*p*-adjusted < 0.05, log2FC ≥ 1.0) in the ovaries of tumor-bearing rats treated with tamoxifen is visualized in Figure 2. The expression profile of the top 100 up- and down-regulated DEGs (i.e., DEGs with the highest and the lowest log2FC values) is presented in Figure 3. The log2FC values for DEGs ranged from −6.03 (Cpn1, carboxypeptidase N subunit 1) to 5.59 (Habp2, hyaluronan binding protein 2) (Table S3).

### 2.2. Functional Enrichment of the Identified DEGs

**Figure 4:**
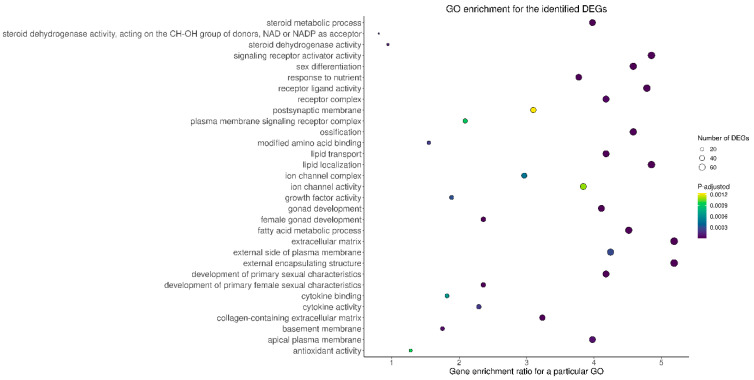
Gene Ontology (GO) analysis of DEGs identified in the ovaries of rats treated with TAM. The *x*-axis represents genes ratio enriched for a particular GO term, while the *y*-axis the names of particular GO terms. The size of the circle denotes the number of DEGs in the term and the circle color represents the range of log10 (*p*-adjusted + 1).

To examine the possible significance of the identified DEGs in the ovaries collected from TAM-treated rats in comparison to control rats, genes were assigned to three main categories within the GO database: (“biological processes” (BP), “cellular components” (CC), “molecular function” (MF)). Seven hundred and seventeen out of 1788 DEGs were ascribed to 711 GO terms (*p*-adjusted < 0.05) including 564 terms within BP, 50 terms within CC and 97 terms within MF categories (Figure 4; Table S4). Within the BP category, the DEGs were mainly enriched in processes related to the development of reproductive functions, and steroid biosynthetic and metabolic processes as well as to lipid localization and transport. Within the CC category, the most DEGs were assigned to extracellular matrix and receptor complexes. Within the MF category, the DEGs were ascribed mainly to receptor and channel activity (Table S4).

**Figure 5:**
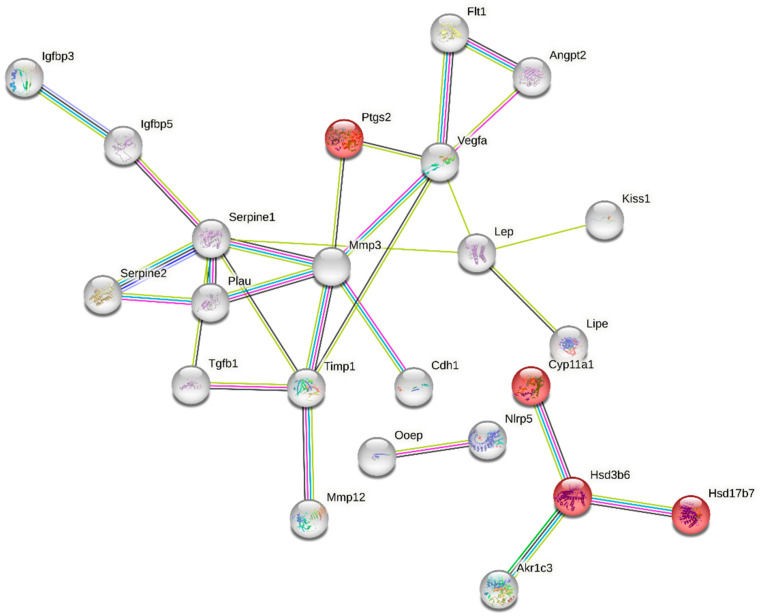
Interaction network of differentially expressed genes (DEGs) identified in the ovaries collected from rats treated with tamoxifen vs. untreated rats. The network was generated by STRING (confidence score: 0.7) using DEGs (*p*-adjusted < 0.05 and log2 fold change ≥ 1.0) belonging to the GO “multi-organism reproductive process” term (GO:0044703). Enrichment *p*-value: 1.0 × 10^−16^. The red nodes represent DEGs related to KEGG pathway “rno04913:Ovarian steroidogenesis” (selected by STRING). The color of an edge is determined by a type of interaction between particular nodes (DEGs): blue—known interactions from curated databases; pink—known interactions experimentally determined; navy blue—predicted interactions gene co-occurrence; green—predicted interactions gene neighborhood; yellow—text mining; violet—protein homology.

The “multi-organism reproductive process” category is closely related to ovarian function and was one of the enriched GO terms containing 49 DEGs (Table S4). Functional classification of these genes performed with the use of STRING (v11.5) produced a gene interaction network with 49 nodes and 28 edges (protein–protein interaction [PPI] enrichment *p*-value: 1.0 × 10^−16^; Figure 5). Nodes devoid of any interactions were deleted from the network. The created network includes genes related to ovarian steroidogenesis (Akr1c3, Ptgs2, CYP11a1, Hsd3b6, Hsd17b7) and cellular response to follicle-stimulating hormone stimulus (CYP11a1, Serpine 1, Akr1c3). We also identified a group of DEGs shown to be associated with apoptosis (e.g., Igfbp3, Serpine 1, Akr1c3, Angpt2, Vegfa, Ptgs2, Lep, Cdh1, Timp1, Tgfb1) (Figure 5, Table S4).

**Figure 6:**
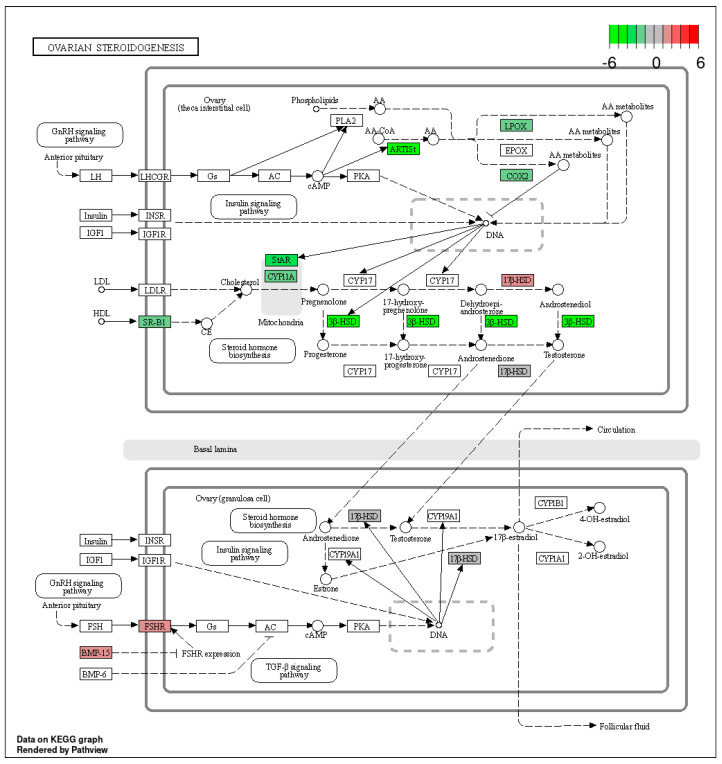
The KEGG “ovarian steroidogenesis” pathway comprising DEGs identified by RNA−Seq in the ovaries of TAM−treated rats. Red and green blocks represent up− and down−regulated DEGs, respectively.

The KEGG database was used to classify the identified DEGs (*p*-adjusted < 0.05) by function, particularly as related to ovarian steroidogenesis—one of the most important aspects of ovarian physiology. Within this pathway, we identified four genes with expression up-regulated after TAM treatment (17β-HSD, Hsd17b2, Fshr and BMP-15) and 13 genes with expression down-regulated (Figure 6, Table S5).

### 2.3. Effects of TAM on the lncRNA Expression Profile of Rat Ovaries

**Figure 7:**
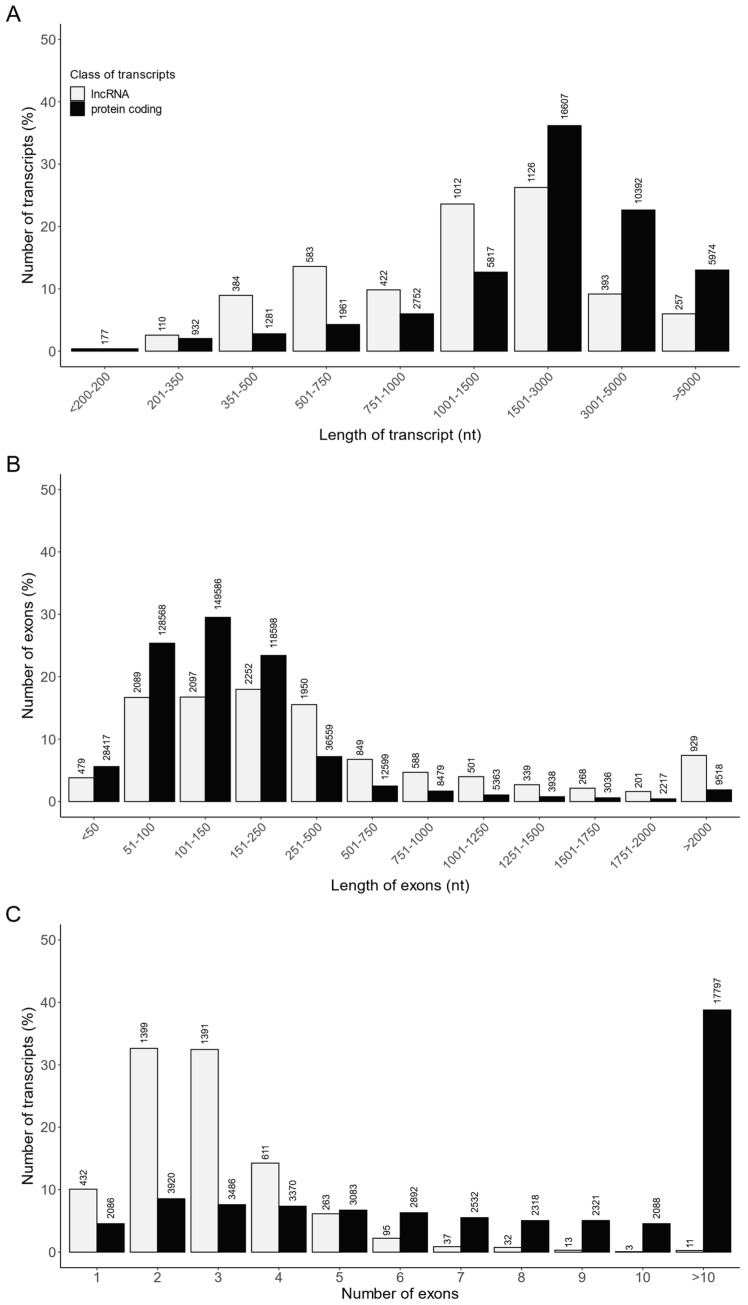
The comparison of the identified lncRNAs and protein coding transcripts according to their (**A**) transcript length, (**B**) exon length and (**C**) exon number.

A customized multistep identification pipeline was applied to distinguish lncRNAs from all assembled transcripts. A total of 4287 RNA sequences were identified as lncRNAs, including 4122 lncRNAs already annotated in the Ensembl database and 165 novel lncRNAs. The transcript length, exon length and exon number were compared between the identified lncRNAs (4287 transcripts) and mRNAs (45,893 transcripts; Table S6, Figure 7). The length of most of the lncRNAs and lncRNA exons ranged from 500 to 3000 nt and from 50 to 500 nt, respectively (Figure 7A,B). In turn, a majority of lncRNAs consisted of two or three exons (Figure 7C). Moreover, the mean length of lncRNAs (1878.6 ± 2053 nt) was shorter (*p* < 0.05) than that of protein coding transcripts (2870.6 ± 2345 nt) (Table S6), and the average exon length of lncRNAs (642 ± 1203 nt) was longer (*p* < 0.05) than that of protein coding (260 ± 545 nt) (Table S6). In addition, the mean exon number of lncRNAs per transcript (2.92 ± 1.44) was lower (*p* < 0.05) than the mean exon number of protein coding transcripts (11.04 ± 10.73) (Table S6).

We identified 243 DELs (log2FC  ≥  1 and *p*-adjusted ≤ 0.05) (Table S7). The log2FC values for DELs ranged from –4.499 (MSTRG.16924) to 3.297 (MSTRG.10313). A total number of 128 DELs identified in the ovaries were up-regulated and 115 DELs were down-regulated by TAM.

**Figure 8:**
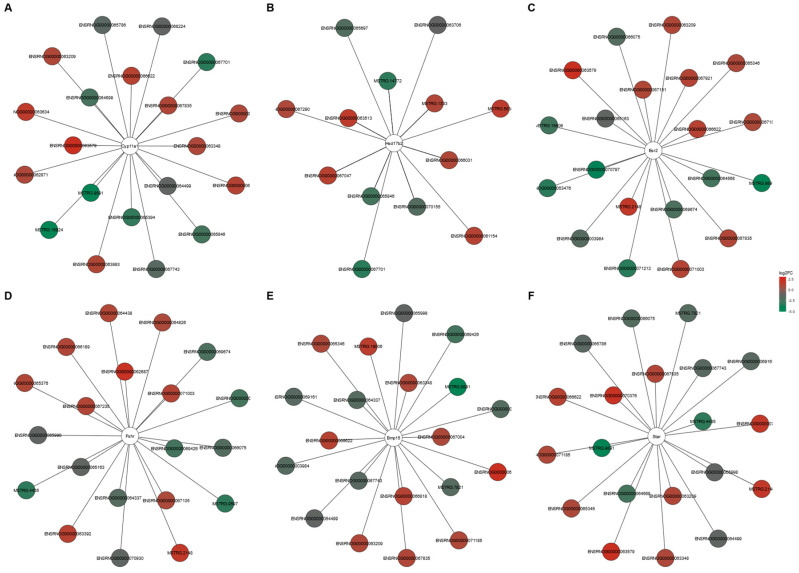
DELs and DEGs co–expression network analysis. Top 10 positive and negative correlations of DELs with the six selected DEGs (**A**–**F**) related to steroid hormone synthesis and metabolism (Pearson correlation coefficient > 0.99). Red nodes and green nodes represent the significantly up–regulated and down–regulated DELs in the ovaries of tumor–bearing rats treated with tamoxifen.

The potential target genes for DELs acting in a *cis*- or *trans*-regulatory manner were predicted to investigate the possible significance of these lncRNAs in the ovarian response to TAM. In silico analysis produced *cis*-target genes for 46 DELs (Table S8), and four of these genes were enriched (*p* < 0.05) in five GO terms of the GO classification (Table S9). As a result of *trans*-type analysis, a total of 19,978 negative and 23,284 positive correlations were detected in the ovaries of tumor-bearing rats treated with TAM (Table S10). For 122 DELs, target DEGs were predicted to be potentially *trans*-regulated by these DELs (Table S11). The negatively co-expressed genes were enriched in 135 GO terms (112 under BP, five under CC and 18 under MF) including “steroid metabolic process” (GO:0008202), “gonad development” (GO:0008406) and “protein maturation” (GO:0051604) (Table S12). The negatively co-expressed *trans*-target genes involved, among others: StAR, Hsd17b2, Dhcr7, CYP11A1, Hsd3b and Hsd3b6 (see Table S12; Figure S1). The positively co-expressed genes were enriched in 195 GO terms (138 in BP, 26 in CC and 31 in MF). Some of the genes were related to “epithelial cell proliferation” (GO:0050673), “hormone transport” (GO:0009914) and “hormone secretion” (GO:0046879) (Table S13). This group of *trans*-target genes involved: Esr2, Pgr, Fshr, Fgf1, Fgf9 and Fgf18 as well as Flt1 (see Table S13; Figure S2). The top 10 positive and top 10 negative correlations of DELs with the six DEGs related to the steroid hormone synthesis and metabolism (StAR, CYP11A1, Hsd17b2, Esr2, Fshr, BMP15) are presented in Figure 8.

### 2.4. Validation of RNA-Seq Data by Real-Time PCR

To validate the RNA-Seq results, five DEGs that were markedly altered by TAM were chosen for real-time PCR analysis: Klk7 (log2FC: 3.19), Prss32 (log2FC: 4.00), Pla2g1b (log2FC: −3.83), Rln1 (log2FC: −5.11) and Amh (log2FC: 1.15). The expression of the selected DEGs obtained by real-time PCR confirmed the results obtained by RNA-Seq (Figure S3).

### 2.5. Effects of TAM on the Proteome of Rat Ovaries

A DIGE-based proteomic approach was used to identify differentially expressed protein spots (DEPSs) in the ovaries of tumor-bearing rats treated with TAM. A total of 959 protein spots were detected on all gels, and 578 of the protein spots were successfully matched between the gels obtained from TAM-treated and control ovaries. A representative gel is presented in Figure S4. The abundance of 14 spots (DEPSs) differed significantly (*p* < 0.05; fold change > 1.5) between CONT and TAM groups. These proteins were used for MALDI-TOF/TOF MS analysis and 11 of them were identified, including vimentin (Vim), prohibitin (Phb), heat shock cognate 71 kDa protein (Hsp7c), mitochondrial aldehyde dehydrogenase (Aldh2), fructose-bisphosphate aldolase A (Aldoa), cytoplasmic actin 1 (Actb), phosphoglycerate kinase 1 (PGK1), calretinin (CALB2), 60 kDa heat shock protein, mitochondrial (CH60), tropomyosin alpha-3 chain (TPM3) and glucose-6-phosphate 1-dehydrogenase (G6PD). Four of the identified proteins (PGK1, CALB2, CH60 and G6PD) were regulated by TAM in the same manner as the genes encoding these proteins (Table 1 and Table S3).

## 3. Discussion

We showed previously that TAM does not affect the number of primordial/primary follicles or the occurrence of granulosal apoptosis in the ovaries of tumor-bearing rats, but it does protect these follicles from the toxic ovarian side effects of the chemotherapeutic agent CPA [17]. In the present study, we explored the ovarian mechanism of TAM action in the same rat model of mammary cancer. Using transcriptomics and proteomic techniques, we identified 1788 DEGs, 243 DELs and 14 DEPSs in the ovaries of TAM-treated animals. Many of these molecules were enriched in GO categories associated with steroid hormone synthesis and metabolism, lipid localization and transport, and epithelial cell proliferation as well as sex differentiation.

Physicians have been using TAM in endocrine therapy for patients with hormone-dependent breast cancer for many years [22], and patients often receive TAM for 5–10 years after the initial treatment to prevent recurrent disease. Despite its well-known efficacy in cancer treatment and proven safety, TAM can induce ovarian hyperstimulation, which may lead to the formation of ovarian cysts and supraphysiological serum estradiol (E_2_) levels [23]. However, most premenopausal women receiving TAM during chemotherapy experience restored menstrual cycles within 5 years [24]. Long-term usage of TAM may contribute to the development of hepatic lipidosis [25,26]. Other side effects attributed to TAM may include hot flushes, vaginal dryness and sleep disorders [27]. Although the effects of TAM therapy have been extensively studied for many years, the available data relate primarily to breast cancer tissue [2,5,22,24]. The impact of TAM on ovarian functions, particularly with respect to ovarian follicle reserves, has not been well studied in breast cancer patients.

In the current study, performed on tumor-bearing rats, we demonstrated that TAM affected the expression of genes and lncRNAs involved in ovarian steroidogenesis (Figure S5). Biosynthesis of steroid hormones is dependent on the availability of StAR (steroidogenic acute regulatory protein), a protein that regulates cholesterol transfer within the mitochondria [28], (the rate-limiting step in the synthesis of steroid hormones) and the activity of the steroidogenesis enzymes P450scc and 3βHSD. Cytochrome P450 side-chain cleavage enzyme (P450scc; *CYP11A1*) converts cholesterol to pregnenolone (P5). 3β-hydroxysteroid dehydrogenase/Δ^5−4^isomerase (3βHSD, *Hsd3b*) converts P5 to progesterone (P4) and 7α-hydroxy-P5 to 17α-hydroxy-P4 and dehydroepiandrosterone (DHEA) to androstenedione (A4) [29]. We found that ovarian genes coding StAR (*STAR*), P450scc (*CYP11A1*) and 3βHSD (*Hsd3b1, Hsd3b2*) were significantly down-regulated by TAM in the current study. These enzymes catalyze the successive steps leading to the synthesis of progestagenes (P5, P4), androgens (DHEA, A4, testosterone [T]) and estrogens (E2, estrone [E1])—hormones that are very important in the regulation of reproduction in females [30]. Moreover, the expression of genes encoding some types of 17β-hydroxysteroid dehydrogenases/17-ketosteroid reductases (*Akr1c2, Akr1c3*) were also decreased by TAM.

These data indicate that TAM inhibited ovarian steroidogenesis in our model of tumor-bearing rats. TAM similarly decreased steroid hormone secretion by blocking the preovulatory LH and FSH surges in cancer-free rats [31]. The effects of TAM on the ovary differ in premenopausal and postmenopausal women since TAM-induced ovarian hyperstimulation and ovarian cysts were observed only in young women (<40 years) [10,23]. Therefore, it seems that the TAM-induced changes in ovarian steroidogenesis are dependent on the species and endocrine status of the female.

We demonstrated previously that TAM alone did not affect the number of ovarian follicles in tumor-bearing rats, but it did prevent the CPA-induced follicular (primordial and primary follicles) loss in these rats [17]. The number of primordial follicles is established before (humans) or around (rodents) birth and serves as the ovarian reserve for future female fertility [32]. Activation of primordial to primary follicles is strictly regulated, and any abnormality during this process can result in pathology. We found in the current study that TAM affected the expression of genes involved in signaling pathways related to primordial follicle activation or arrest (Figure S5). The expression of the inhibin α (*Inha*) and anti-Mullerian hormone (*Amh*) genes was up-regulated by TAM alone (the current study) as well as during ovotoxic chemotherapy [17]. These genes are down-regulated during the normal primordial to primary follicle transition in rats [33]. Amh is produced exclusively during the early stages of follicle development [32], supporting the notion that TAM maintains the follicular reserve in females [17,20]. Moreover, in the current study, TAM induced ovarian expression of estrogen receptor β (*Esr2*), and this gene plays an essential role in the recruitment of growing follicles. Knockout of *Esr2* resulted in the increased activation of primordial follicles in rats [34]. This suggests that TAM can inhibit the premature activation of primordial follicles—an established mechanism of follicular loss [32]. Additionally, genes encoding steroidogenesis enzymes (*CYP11A, Hsd3b1, Hsd3b2, Akr1c2, Akr1c3*) as well as StAR protein, inhibited by TAM in the current study, were found to be up-regulated during the transition between the unassembled (oocyte nests) and primordial follicles stages [33]. Exploration of this promising action of TAM appears critical.

The abundance of 14 protein spots significantly differed between the ovaries of TAM-treated and control rats; 11 of these spots were successfully identified in the current study. Specifically, TAM down-regulated the abundance of CH60, Vim, CALB2, PGK1, G6PD, TPM3, Aldoa and Phb proteins and up-regulated Actb, Aldh2 and Hsp7c. The expression of PGK1, CALB2, CH60 and G6PD proteins was also down-regulated at the mRNA level. PGK1 is the first ATP-producing enzyme in glycolysis; therefore, it plays a key role in cellular redox balance, and was recently proposed as a molecular target for antiglycolytic therapy of ovarian cancer [35]. G6PD is crucial for NADPH generation, and down-regulation of G6PD may enhance tumor sensitivity to chemotherapeutic drugs [36]. CALB2, in turn, is a calcium-binding protein expressed in theca interna and other cells; the protein was down-regulated in the ovaries of rats following exposure to antiandrogenic chemicals [37]. Finally, CH60 is a chaperonin suggested to regulate cell cycle and apoptosis in cancer cells. CH60 overexpression has been observed in tertiary and atretic follicles [38]. One limitation of the current proteomic experiment is the sensitivity of the gel-based approach. However, the identified proteins affected by TAM in the current study offer new knowledge on the molecular mechanism of TAM action in the ovary.

The results of the present study show that the molecular mechanisms of TAM action in the ovaries of tumor-bearing rats relate to signaling pathways responsible for steroid hormone synthesis and inhibition of primary and primordial follicle activation as well as basic metabolic processes. Although simple extrapolation of these data to humans is not possible, our results emphasize the need to explore TAM and its ability to affect ovarian function in women undergoing cancer treatment. TAM remains a mainstay for both the treatment and prevention of breast cancer in women. Preclinical studies from our group and others suggest a novel protective ovarian action of TAM during cancer treatment. Therefore, research concerning the mechanism of TAM action in the ovaries of females with cancer is of great importance. Further investigation on the ovarian mechanism of TAM will require in-depth functional studies.

## 4. Material and Methods

### 4.1. Animals and Treatments

The protocol of the study was approved by the Local Ethics Committee for Animal Experiments in Olsztyn, Poland (decision No. 78/2017/WNP). Female Wistar rats were housed in a controlled environment (22 °C; 60% humidity; 12L:12D) in the Center of Experimental Medicine (Bialystok, Poland) with ad libitum access to food and water. The experiment was performed on rats with MNU-induced mammary neoplasia [17]. Rats were randomly assigned to two groups: (1) control group (CT, control group) and (2) tamoxifen (TAM)-treated group. On day 1 of the experiment, TAM rats received subcutaneous implants gradually releasing tamoxifen (1 mg/kg b.w./day; Innovative Research of America, Sarasota, USA; [39,40]) and control rats received placebo implants. All rats were sacrificed on day 34 of the experiment, ovaries were collected, snap frozen in liquid nitrogen and stored in −80 °C for future analyses (RNA-Seq, 2D-DIGE + mass spectrometry, MS).

### 4.2. Total RNA Isolation and Sequencing

Total RNA was isolated from ovaries (n = 4 rats/group) using peqGold TriFast reagent. RNA concentration and quality were determined spectrophotometrically (NanoVue Plus, GE Healthcare, Little Chalfont, UK). To evaluate RNA integrity, a microfluidic electrophoresis (2100 Bioanalyzer; Agilent Technologies, Santa Clara, CA, USA) was employed. Samples with an RNA integrity number (RIN; 28 S/18 S ratio) of at least 8.0 were used for RNA-Seq performed by Macrogen (Seoul, Republic of Korea). Total RNA was used to construct cDNA libraries (TruSeq stranded mRNA Sample Preparation Kit, Illumina, San Diego, CA, USA). The libraries were prepared by random fragmentation of cDNA samples followed by 5′ and 3′ adapter ligation. Adapter-ligated fragments were amplified (PCR). The cDNA library templates were then loaded into the flow cells, where fragments were captured on a lawn of surface-bound oligos complementary to the library adapters. Each fragment was then amplified into a distinct clonal cluster through bridge amplification. After cluster generation was completed, the cDNA templates were designated for sequencing. A NovaSeq6000 high-throughput sequencing instrument (Illumina) was used for 100 bp paired-end configuration sequencing.

Results of the RNA-Seq revealed that one TAM sample (TAM 4) displayed a transcriptome profile different from TAM 1, 2 and 3 samples. This sample was excluded from further analysis. Thus, the entire analysis of transcript expression level was performed on four control samples and three TAM-treated samples.

### 4.3. Identification of lncRNA

The quality of raw reads was evaluated by means of FASTQC (https://www.bioinformatics.babraham.ac.uk/projects/fastqc/, accessed on 1 September 2022). The low-quality reads and adapters were removed with the use of Trimmomatic (version 0.39; https://doi.org/10.1093/bioinformatics/btu170, accessed on 1 September 2022). The obtained reads were mapped to the rat reference genome (mRatBN7.2; Ensembl release 107, accessed on 2 September 2022) using STAR software (version 2.7.10a; https://doi.org/10.1093/bioinformatics/bts635, accessed on 2 September 2022). The mapped reads were assembled into transcripts by StringTie (version 2.2.1; https://doi.org/10.1038/nbt.3122, accessed on 2 September 2022). The assembled transcripts were compared with reference annotation using Gffcompare (version 0.12.6; https://doi.org/10.12688/f1000research.23297.1, accessed on 5 September 2022). To predict novel lncRNAs, transcripts with class code “x”, “o”, “i”, “u” and “j” were retained [41]. Next, the transcripts with length < 200 nt and exon number < 2 were removed as a potential bias, and coding potential of the remained transcripts was evaluated by means of TransDecoder (version 5.5.0, accessed on 5 September 2022). Transcripts with open reading frame (ORF) < 300 nt (100 a.a.) were excluded from the analysis. Afterwards, the random forest (RF) classifier was used to create a mathematical model to predict lncRNA transcripts. Briefly, nucleotide sequences of mRNA and lncRNA for human (GRCh38) and rat (mRatBN7.2) were downloaded from the Ensembl database (release 107, accessed on 2 September 2022). For both TAM-treated and control groups, transcripts with identical nucleotide sequences were deduplicated as a potential bias. Next, LncFinder 1.1.4 was employed to extract features for each nucleotide sequence (https://doi.org/10.1093/bib/bby065, accessed on 6 September 2022). These features were used to create and train RF classifier using 10-fold cross-validation by means of scikit-learn 1.1.2 (accessed on 7 September 2022). The precision and recall of the RF model during the hyperparameters’ tuning were evaluated on a test dataset. This model was used to predict lncRNAs, and transcripts classified as mRNA were removed from further analysis. Transcripts predicted to be potential lncRNAs were used to search orthologs employing BLASTn (version 2.13.0; https://doi.org/10.1186/1471-2105-10-421 (accessed on 26 October 2023)) against the NCBI Nucleotide Database (accessed on 20 October 2022) with E-value threshold set to 10^−5^. Based on the BLASTn results, transcripts were classified into three groups: (1) transcripts with significant BLASTn match to protein coding gene or non-lncRNA transcripts; (2) transcripts with significant BLASTn match to lncRNA gene; and (3) transcripts with no significant BLASTn match. Transcripts classified to the first group were excluded from the analysis, while transcripts from the second and third groups were used for further analysis as novel lncRNAs.

### 4.4. Identification of Differentially Expressed lncRNAs (DELs) and Genes (DEGs)

After identification of lncRNAs, raw counts per genes were calculated by featureCounts (version 2.0.3; https://doi.org/10.1093/bioinformatics/btt656, accessed on 21 October 2022). The differentially expressed lncRNAs (DELs) and genes (DEGs) as well as corresponding *p*-adjusted values were determined by means of R statistical software (version 4.2.1., accessed on 21 October 2022) using the DESeq2 package (version 1.36.0; https://doi.org/10.1186/s13059-014-0550-8, accessed on 26 October 2023). The threshold for the significantly different expression was set at *p*-adjusted ≤ 0.05 and log2 fold change (log2FC) ≥ 1.0 or log2FC ≤ −1.0. The visual presentation of the results was performed by R software using the ggplot2 (version 3.3.2, accessed on 21 October 2022) and gplots (version 3.3.6; ISBN 978-0-387-98141-3, accessed on 21 October 2022) packages.

### 4.5. Cis- and Trans-Regulated Target Gene Prediction of lncRNAs

To explore *cis-* and *trans*-type interactions, the Pearson’s correlation coefficient between expression profile for DEGs and DELs was determined by means of the Hmisc package (version 4.6-0) implemented into R statistical software. For *cis*-type interactions, only DEGs located with a distance less than 20 kb from DELs were screened. The threshold for the DEL-regulated DEGs was set as *p*-value ≤ 0.05 and Pearson’s correlation coefficient r ≥ 0.90 or r ≤ −0.90.

### 4.6. Functional Enrichment Analysis

Functional analyses of both the identified DEGs and the DEGs targeted by DELs were performed based on the Gene Ontology (GO) database using the clusterProfiler (version 4.4.4; https://doi.org/10.1089/omi.2011.0118, accessed on 24 October 2022), DOSE (version 3.22.1; https://doi.org/10.1093/bioinformatics/btu684, accessed on 24 October 2022), biomaRt (version 2.52.0; https://doi.org/10.1038/nprot.2009.97, accessed on 24 October 2022) and org.Rn.eg.db (version 3.15.0; https://doi.org/10.18129/B9.bioc.org.Ss.eg.db, accessed on 24 October 2022) packages of R software, with the established criterion *p*-adjusted ≤ 0.05. Additionally, the Kyoto Encyclopedia of Genes and Genomes (KEGG database, accessed on 24 October 2022) was used to ascribe identified DEGs and lncRNA targets to particular biological mechanisms and cellular pathways (*p*-adjusted ≤ 0.05). The KEGG enrichment analysis was performed by the clusterProfiler, DOSE and org.Rn.eg.db packages of R software. The visual presentation of the results was performed by R software using ggplot2. In addition, the Bioinformatics Database STRING 11.5 (Search Tool for the Retrieval of Interacting Genes, http://string-db.org, accessed on 24 October 2022) was used to explore possible networks for the identified DEGs [42]. The searching criteria were based on the occurrence of genes/proteins in scientific texts (text mining), co-expression and experimentally observed interactions. This analysis generated gene/protein interaction networks, where the strength of the interaction score was set as 0.7.

### 4.7. Real-Time PCR

Real-time PCR was used to validate the results of RNA-Seq by measuring the expression of five selected DEGs identified in the control and experimental ovaries (n = 4 per group). The RT reaction and real-time PCR were performed as previously reported [43]. Primers and probes (Thermofisher Scientific, Waltham, MA, USA) for particular genes are presented in Table S1.

### 4.8. Protein Isolation and 2D-DIGE

Rat ovarian proteins (n = 6 rats/group) were extracted with lysis buffer (7M urea, 2% *w*/*v* CHAPS, 2% ampholytes (pH 4–7NL; GE Healthcare, Chicago, IL, USA), 120 mM dithiothreitol, protease inhibitors cocktail (Sigma Aldrich, St. Louis, MO, USA), 0.002% bromophenol blue). The isolation and purification procedures were performed as previously described [44]. The protein concentration was determined before and after purification, using a 2D-PAGE adaptation of the Bradford assay [45] with BSA dissolved in rehydration buffer (7M urea, 2M thiourea, 2% CHAPS, 130 mM DTT, 2% ampholytes (pH 4–7 NL)) as a protein standard. BSA standards and the examined samples were acidified with 10 μL of 0.1M HCl. The measurements were carried out at a wavelength of 595 nm using an Infinite M200 multimode microplate reader (Tecan, Grodig, Austria). The obtained protein extracts were used in 2D-DIGE.

The protein extracts (50 μg) from each sample (n = 6 ovaries/group) were dissolved in labelling buffer (30 mM Tris, 7M urea, 2M thiourea, 4% *w*/*v* CHAPS, pH 8.0), and labelled with CyDye DIGE Fluor minimal dyes (GE Healthcare, reconstituted in fresh 99.8% anhydrous dimethylformamide) at a concentration of 400 pmol dye/50 μg of protein. The labelling was performed in the dark (30 min, on ice) to avoid photobleaching of the fluorescent dyes. Differentially labelled proteins (Cy2-, Cy3-, Cy5-labelled) were mixed. A dye swap of control and TAM-treated samples was performed to exclude dye bias. The rehydration and separation by isoelectric focusing were performed as previously described [44]. The second dimension (SDS-PAGE) was performed using 12.5% SDS polyacrylamide gels in the Ettan DALTsix electrophoretic unit (GE Healthcare) at 20 °C (1.5 W/gel for 16 h). To visualize the spots, the gels with separated proteins were scanned with an Ettan DIGE Imager (GE Healthcare). Image analysis was performed using SameSpots software (Totallab, Newcastle, UK). The calculated volume of each spot was normalized against the volume of the Cy2 labelled internal standard spot. In order to investigate changes in the proteome induced by TAM, the spots derived from the control and TAM samples were matched. The spots with significant abundance changes (*p* < 0.05 and fold change ≥ 1.5) between the control and TAM-treated samples (differentially expressed protein spots; DEPSs) were designated to MS for protein identification.

### 4.9. Protein Digestion and MALDI-TOF/TOF Analysis

The gels were re-stained using Coomassie Brilliant Blue G-250 (BioRad, Hercules, CA, USA), and the DEPSs from 2D separations were dissected from the gels. Proteins in these spots were digested, and MS analysis (MALDI-TOF/TOF) was performed as previously described [44]. Statistical probability of the correct prediction of the identified protein (including peptide mass fingerprint and ion scores) was calculated by MASCOT software. Scores above 70 (*p* < 0.05) were considered significant.

## Figures and Tables

**Table 1:** Differentially expressed proteins identified in the ovaries of mammary-tumor-bearing rats treated with tamoxifen vs. control rats.

Identified Proteins	MASCOT Protein Score	Sequence Coverage (%)	Number of Peptides	Fold Change	Accession Number
60 kDa heat shock protein, mitochondrial (CH60)	228	29	11	−4.1	gi ǀ206597443
Vimentin (VIM)	287	26	12	−2.3	gi ǀ38197662
Calretinin (CALB2)	113	14	4	−2.1	gi ǀ16758892
Phosphoglycerate kinase 1 (PGK1)	52	3	1	−1.8	gi ǀ56585024
Glucose-6-phosphate 1-dehydrogenase (G6PD)	69	12	5	−1.8	gi ǀ8393381
Tropomyosin alpha-3 chain (TPM3)	722	49	22	−1.8	gi ǀ669633260
Fructose-bisphosphate aldolase A (ALDOA)	108	16	4	−1.7	gi ǀ408772019
Prohibitin (PHB)	83	21	5	−1.4	gi ǀ13937353
Actin, cytoplasmic 1 (ACTB)	105	35	1	2.5	gi ǀ13592133
Aldehyde dehydrogenase, mitochondrial (ALDH2)	263	16	8	2.3	gi ǀ1820958497
Heat shock cognate 71 kDa protein (HSP7C)	107	10	5	1.9	gi ǀ13242237

## Data Availability

All relevant data are within the paper and its Supplementary Materials, and in the BioProject database under accession number: PRJNA640997.

## Supplementary Materials

The following supporting information can be downloaded at: https://www.mdpi.com/article/10.3390/ijms242115767/s1.
