# Supplementary material for: Molecular Action of Tamoxifen in the Ovaries of Rats with Mammary Neoplasia
Source: Int J Mol Sci. 2023 Oct 30;24(21):15767. doi: 10.3390/ijms242115767 (PMC10649132; doi:10.3390/ijms242115767)
Supplement: Supplementary file 1 [file ijms-24-15767-s001.zip › supplementary/Supplementary Materials captions.pdf]

Supplementary Materials:

Figure S1: Interaction network of differentially expressed genes (DEGs) identified as negatively regulated by DELs in the ovaries collected from rats treated with tamoxifen *vs.* untreated rats. The network was generated by STRING (confidence score: 0.7) using DEGs (P-adjusted < 0.05 andlog2 fold change ≥1.0) belonging to the GO "steroid metabolic process" term (GO:0008202). Enrichment P-value: 1.0×10^{-16};

Figure S2: Interaction network of differentially expressed genes (DEGs) identified as positively regulated by DELs in the ovaries collected from rats treated with tamoxifen *vs.* untreated rats. The network was generated by STRING (confidence score: 0.7) using DEGs (P-adjusted < 0.05 andlog2 fold change ≥1.0) belonging to the GO "epithelial cell proliferation" term (GO:0050673). Enrichment P-value: 1.0×10^{-16};

Figure S3: Real-time PCR validation of two selected differentially expressed genes (DEGs) which were identified by RNA-Seq in the ovaries of rats treated with tamoxifen (TAM) (n=4 biological replicates). Data were expressed as arbitrary units (AU; mean±SEM). Statistical analysis was performed using t-student test, *P<0.05;

Figure S4: Representative image (an overlay of Cy3 - green and Cy5 –red channel images) of 2D-DIGE separation of proteins isolated from the ovaries collected from rats treated with tamoxifen *vs*. untreated control rats. Circles depict differentially expressed protein spots and shortcuts describe proteins identified by mass spectrometry;

Figure S5. Tamoxifen (TAM)-induced changes in the expression level of selected differentially expressed genes (DEGs) related to ovarian steroidogenesis (A) and follicle transition (B). Data are expressed as mean of $\log_{10}$(counts+1). Panel C presents the same changes expressed as log2 fold change values. Statistical analysis was performed by means of R statistical software (version 4.2.1.) using the DESeq2 package (version 1.36.0; doi: 10.1186/s13059-014-0550-8). **P-adjusted ≤ 0.01, *** P-adjusted ≤ 0.001;

Table S1: Primers and probes used for real-time PCR;

Table S2: Summary of sequence read alignments to the reference genome;

Table S3: Differentially expressed genes (DEGs) identified in the ovaries of tumor-bearing rats treated with tamoxifen (TAM);

Table S4: Functional enrichment analysis (GO) of the differentially expressed genes identified in ovaries of tumor-bearing rats treated with tamoxifen;

Table S5: KEGG enrichment analysis of the differentially expressed genes identified in ovaries of tumor-bearing rats treated with tamoxifen;

Table S6: The comparison of lncRNAs and protein coding transcripts;

Table S7: Differentially expressed lncRNA (log2FC ≥ 1 and padj⩽0.05) identified in the ovaries of tumor-bearing rats treated with tamoxifen;

Table S8: Target DEGs predicted to be potentially cis-regulated by DELs;

Table S9: Functional enrichment analysis (GO) of the co-expressed cis-target genes predicted to be regulated by differentially expressed lncRNAs identified in the ovaries of tumor-bearing rats treated with tamoxifen;

Table S10: The Pearson correlation between expression patterns of trans-regulated DEGs and DELs identified in the ovaries of tumor-bearing rats treated with tamoxifen;

Table S11: Target DEGs predicted to be potentially trans-regulated by DELs;

Table S12: Functional enrichment analysis (GO) of the negatively correlated co-expressed trans-target genes predicted to be regulated by differentially expressed lncRNAs identified in the ovaries of tumor-bearing rats treated with tamoxifen;

Table S13: Functional enrichment analysis (GO) of the positively correlated co-expressed trans-target genes predicted to be regulated by differentially expressed lncRNAs identified in the ovaries of tumor-bearing rats treated with tamoxifen.
