# Supplementary material for: Molecular Action of Tamoxifen in the Ovaries of Rats with Mammary Neoplasia
Source: Int J Mol Sci. 2023 Oct 30;24(21):15767. doi: 10.3390/ijms242115767 (PMC10649132; doi:10.3390/ijms242115767)
Supplement: Supplementary file 1 [file ijms-24-15767-s001.zip › supplementary/Table S6.pdf]

Supplementary Table S6. The comparison of lncRNAs and protein coding transcripts

Gene type	Number of transcripts	Transcript length (nt)		Exon length (nt)		Exon mumber	
		Mean±SD	Min/Max	Mean±SD	Min/Max	Mean±SD	Min/Max
**lncRNA**	4287	1878.6±2053.3	256/32349	642.1±1204	10/18431	2.93±1.4	1/16
**Protein coding transcripts**	45893	2870.6±2345.5	27/107207	259.9±131.6	1/19890	11.1±10.7	1/349
