# Supplementary figures and images for: Molecular Action of Tamoxifen in the Ovaries of Rats with Mammary Neoplasia
Source: Int J Mol Sci. 2023 Oct 30;24(21):15767. doi: 10.3390/ijms242115767 (PMC10649132; doi:10.3390/ijms242115767)

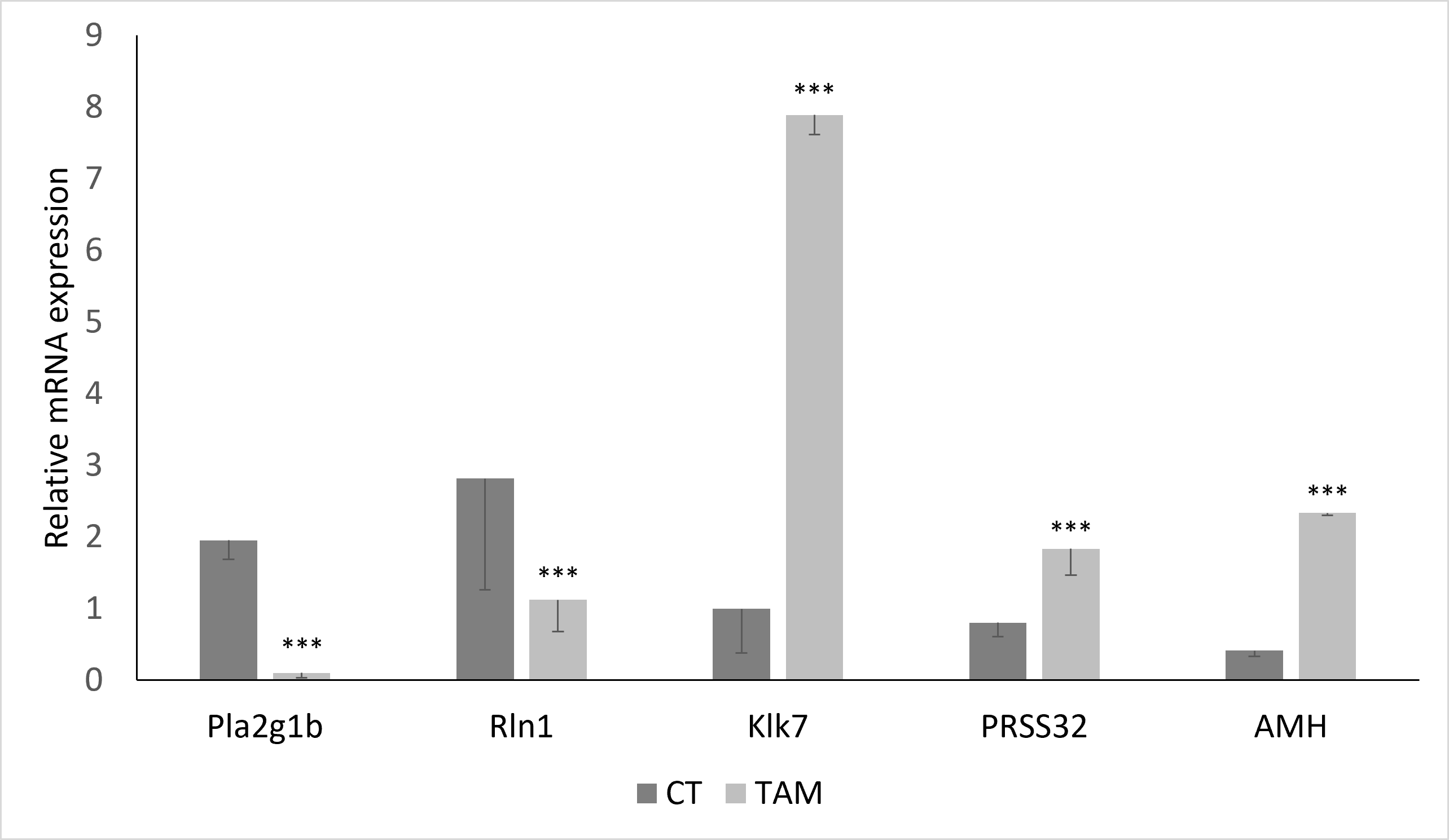

Supplement: Supplementary file 1 [file ijms-24-15767-s001.zip › supplementary/Figure S3.png]

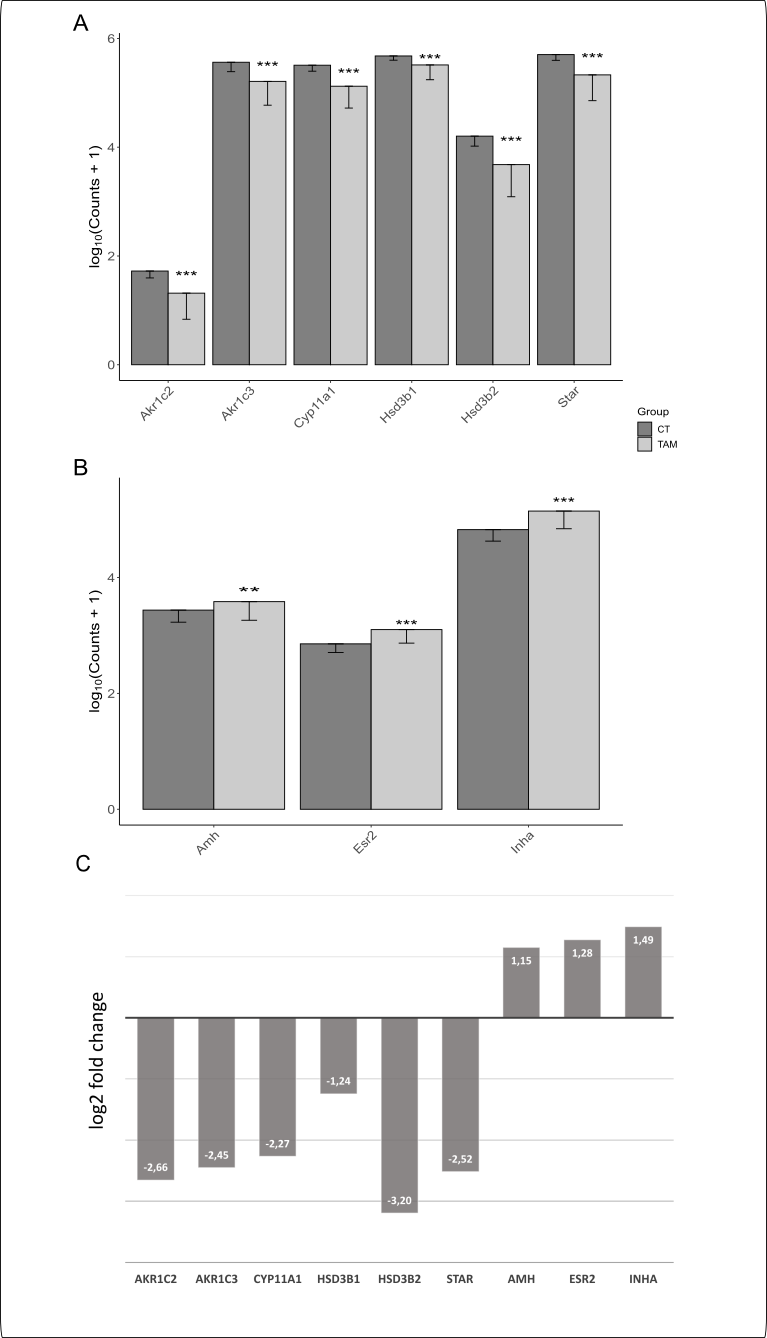

Supplement: Supplementary file 1 [file ijms-24-15767-s001.zip › supplementary/Figure S5.png]

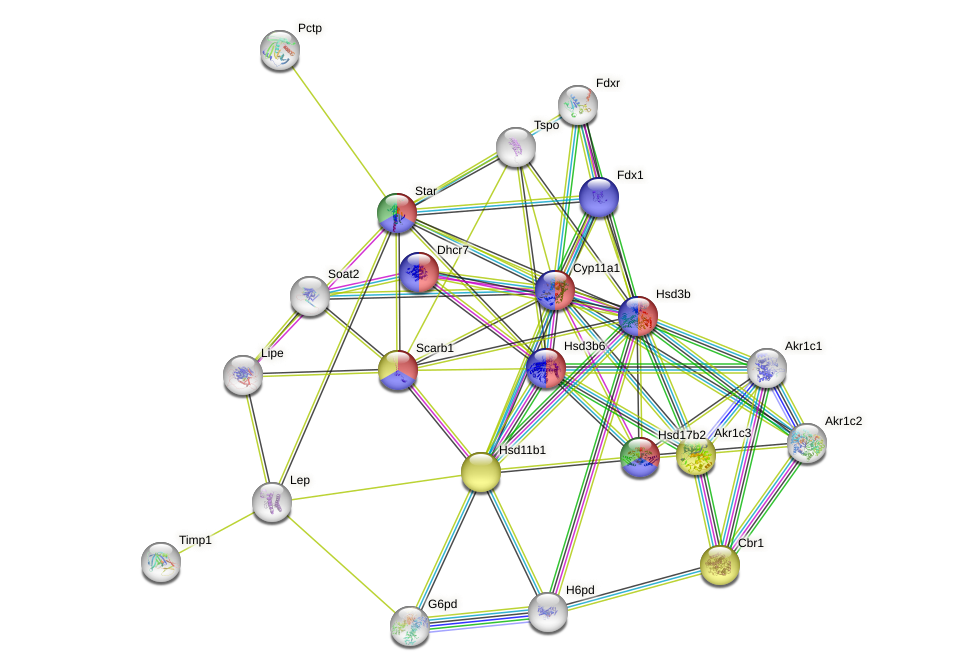

Supplement: Supplementary file 1 [file ijms-24-15767-s001.zip › supplementary/Figure.S1.png]

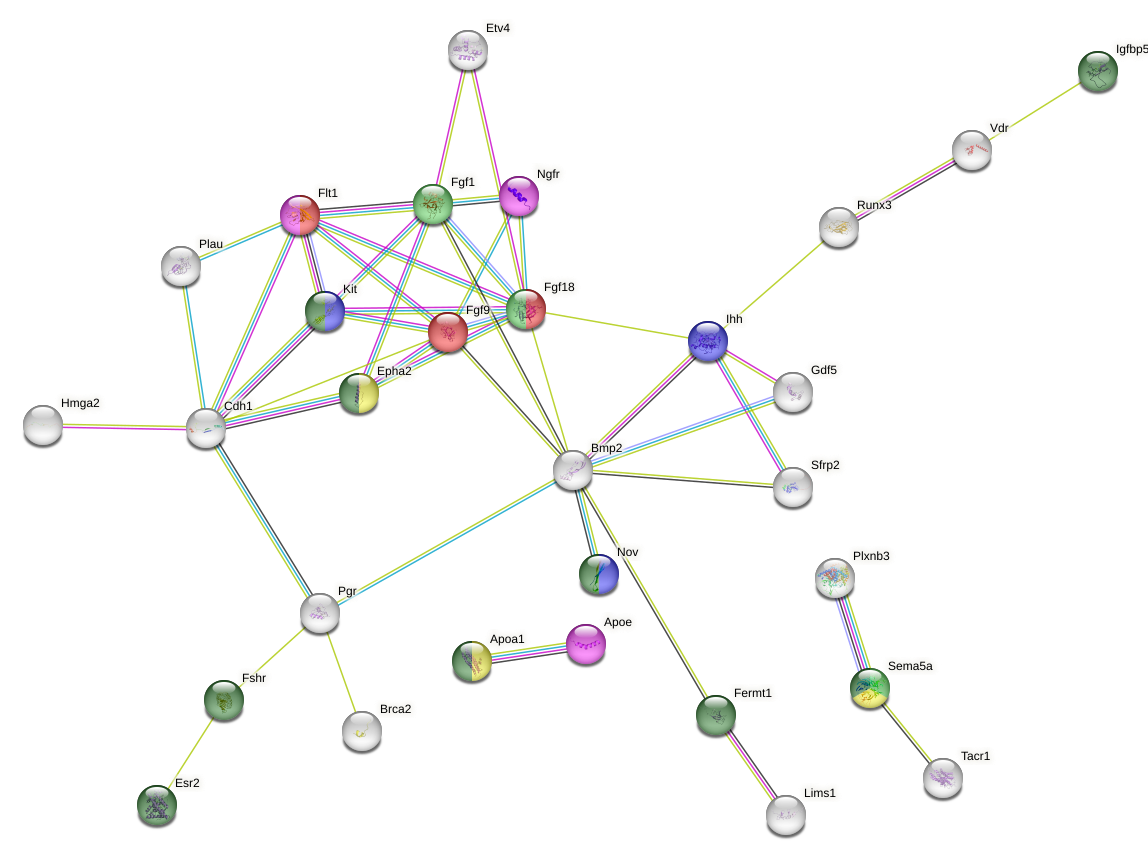

Supplement: Supplementary file 1 [file ijms-24-15767-s001.zip › supplementary/Figure.S2.png]
